# Tanner Put to Open Shame by a Fasting Prisoner

**Published:** 1882-11

**Authors:** K. P. Moore

**Affiliations:** Forsyth, Ga.


### TANNER PUT TO OPEN SHAME BY A FASTING PRISONER.

By K. P. MOORE, M.D, Forsyth, Ga.

When Dr. Tanner was astonishing the world some time ago by his forty days fast, I confess that my faith was not very strong in the genuineness of his pretentions. I thought that it was more than probable that some concentrated preparation of food was being slipped into his "jug of spring water," but I am now prepared to believe the "fish story."

There is now confined in the jail of this county a bright mulatto negro man, 26 years of age, who hails from Greenville, S. C., and who gives his name as Perry J. Cooley. He was tried before a justice of the peace for burglary, and failing to give bond, was committed to jail on the 8th of May, 1882. When he went into jail he was the picture of health; was large, robust and fat. His average weight was 170 pounds, according to his own statement. In a few days he began to complain to Mr. C. A. King, the sheriff and jailer, of being sick, and wanted to take pills. Mr. K. gave him several doses of cathartic pills, and finally, in compliance with his importunities, furnished him a box of twenty-five improved compound cathartic pills. These he took freely for several days, until the janitor complained to Mr. K. that Cooley was purging himself too much. In the mean time Cooley had been begging the sheriff to put him into a cell to himself, and accusing the other prisoners in his cell of abusing him. To pacify him, the sheriff trrnsferred him to a cell by himself. He now at once stuffed pieces of blankets, etc., into every crack and crevice in his room and made it perfectly dark. Mr. King noticed that Cooley was not eating anything, and in speaking of it to his former room-mates was told that he had not eaten anything for some days before he left their room.

This state of affairs continued for several weeks, when Mr. King, becoming a little uneasy for his ward, called in my partner, Dr. L B. Alexander, to see him. Dr. A. made his first visit on the 18th of July. He found Cooley much reduced in flesh and strength; indeed too weak to sit up at all, and with hardly strength enough to speak in an audible voice, but with no indication of disease of any character whatever. He had now gone, according to the best estimates that could be made, since the middle or latter part of May up to the 18th of July without taking food.

By Dr. Alexander's direction, he was moved down stairs and put into a large and well-ventilated room, and ordered for his diet milk punch, soup, etc.

Dr. A. made him one or two more visits and pursued about the same plan of treatment.

As there was no money in the case, and the patient being a prisoner and seeming determined upon self-destruction, he was rather left to follow his own inclinations and to enjoy his right to eat or not, as he might feel inclined. He absolutely and persistently refused to take soup or nourishment in any form, except the milk punch and a little ice and wine. This he got rather sparingly, until about the first of August, when the sheriff began to furnish him about a half pint of wine per day with a little ice. Dr. A. did not see him again until the first week in August, when I was invited by the Doctor to visit the jail with him. We found that Cooley had become exceedingly emaciated, and his strength was entirely gone; his temperature was not more than 95; pulse about 60; and respiration 8. He craved nothing but ice and whisky or wine, but said he would take ice cream. For some days we were quite busy, and did not see him. On August 14th, in the absence of Dr. A., I went with the sheriff to the jail and found that our directions had not been carried out, or, if at all, very imperfectly. The patient had now grown too weak to turn himself, and was barely able to whisper. Blood had settled in large splotches and ecchymosed spots all over his body and limbs; his surface was very cold; temperature below 90; pulse 54; and general condition exceedingly unpromising. Indeed, I felt that he was almost "in articulo mortis," and told Mr. King that without very strict and close attention and careful nursing, he would not live twenty-four hours. I ordered a hot milk toddy every four hours, and having comparative leisure, I determined to see that my orders were strictly observed. With my own hands I assisted in carrying him out into the yard and gave him a sun bath; had him well rubbed with cloths wrung out of hot water, and wrapped in a blanket. He rallied very kindly under this treatment, and the next morning, Dr. A. having arrived, we both went to see him, and told him that he had been left to the enjoyment of his freedom and the exercise of his volition as long as we intended to allow, and that he now had to make up his mind to eat whatever we desired him to take, or we would force a tube into his stomach and pump it into him. Whereupon he submitted to our judgment and agreed to do as we required. We found his general condition to be decidedly better than on the previous day, but still quite critical. We now prepared some beef tea, and with much difficulty he swallowed a teacupful. We put him in charge of a nurse, after moving him into the yard, and urged repeating the beef tea, alternating with milk and whisky. This plan of treatment was pursued for several days with gratifying results. He steadily improved every day, and with returning strength we increased the quantity and quality of his diet. The ecchymosed condition of his skin has gradually faded, and he is now at this writing (August 24th) rapidly recovering.

I regret that we did not look more closely after him, and keep a more accurate account and record of his case from day to day, and that we did not have him weighed occasionally during his fast. I weighed him this morning and found his weight to be 98 pounds. Judging from his appearance at this time I should say that he had gained at least 12 or 15 pounds since we began looking more closely after him. If my suppositions are correct, his weight, when he was taken in hand for treatment, must have been less than half of what it was when he was put into jail. He was a mere living skeleton, the most emaciated person I ever saw. His back and belly looked as if they had literally grown together. The bodies of the spinal vertebræ pressed out the thin abdominal wall in front, and the pulsations of the aorta could be plainly seen. To conclude, I briefly look back over this case and sum up the amount of nourishment taken by Cooley from about the middle of May up to the time that Dr. Alexander first saw him, on the 18th of July. From the best evidence that we can get from Mr King, the sheriff, and by the way, a high-toned, truthful, reliable man, he ate almost nothing. Dr. Alexander thinks it would be quite safe to say that, from the time he first saw him up to the time I saw him, during the first week in August, he had not taken more than one gallon of milk, and perhaps a quart of wine, and not more than a pint of whisky. And up to August 14th, when we decided to force nourishment upon him, he had taken not exceeding two gallons of milk, and we are confident not more than a gallon of wine, and hardly a quart of whisky. So we feel it would be quite safe to say, that he lived three months without taking any solid food at all, and only the amount of liquids mentioned above.

I have read this report to my partner, Dr. Alexander, and he fully endorses it, and says that if I have made an error at all in estimating the amount of food taken by Cooley, I have put it too high. That he is satisfied I have not overdrawn the picture or overestimated the quantity of aliment consumed within the three months.

